# Socioeconomic and geographical inequalities in delivery by cesarean section among women in Bangladesh, 2004–2017

**DOI:** 10.1186/s12884-024-06327-z

**Published:** 2024-02-13

**Authors:** Satyajit Kundu, Azaz Bin Sharif, Syed Sharaf Ahmed Chowdhury, Sadia Afroz, Rakhi Dey, Ahmed Hossain

**Affiliations:** 1 Global Health Institute, North South University, Dhaka, 1229 Bangladesh; 2 Faculty of Nutrition and Food Science, Patuakhali Science and Technology University, Patuakhali, 8602 Bangladesh; 3 Department of Public Health, North South University, Dhaka, 1229 Bangladesh; 4 Statistics Discipline, Khulna University, Khulna, 9208 Bangladesh; 5 College of Health Sciences, University of Sharjah, Sharjah, 27272 United Arab Emirates

**Keywords:** Cesarean section, Delivery, Inequality, Trends, Bangladesh

## Abstract

**Background:**

There is a dearth of evidence on the trends and inequalities in utilizing cesarean section (CS) among women in Bangladesh. Hence, this study aimed to estimate the socioeconomic and geographical inequalities in delivery by CS among Bangladeshi women from 2004 to 2017.

**Methods:**

Data from Bangladesh Demographic and Health Survey 2004, 2007, 2011, 2014, and 2017 were analyzed using the WHO’s Health Equity Assessment Toolkit (HEAT) software. Inequalities were measured using four summary measures: Difference (D), Population Attributable Risk (PAR), Population Attributable Fraction (PAF), and Ratio (R). Socioeconomic inequalities were assessed using two equity dimensions: household wealth status, and level of education, while geographical disparities were measured using two equity dimensions: place of residence, and sub-national regions. For each measure, point estimates and their 95% confidence intervals were reported.

**Results:**

An increasing trend in the prevalence (weighted) of CS in Bangladesh use was found from 4.50% in 2004 to 32.73% in 2017 We found significant socioeconomic inequalities in CS in every survey point, with a higher concentration of CS among the rich (in 2017, PAR = 28.57; 95% CI: 26.69–30.46) indicating a pro-rich inequality, and higher educated (in 2017, PAF = 23.97; 95% CI: 12.26–35.68) sub-groups. We also identified significant geographical disparities in CS with a higher concentration of CS among people from urban areas (in 2017, PAR = 10.99; 95% CI: 10.19–11.79), and a coastal region (Khulna division) (in 2017, PAF: 30.48 (95% CI: 18.66–42.30).

**Conclusion:**

We observed both socioeconomic and geographical inequalities in CS exist in Bangladesh, though the trends of these inequalities were curved over time. Thus, it is important to comprehend these pro-rich and geographical inequalities better and implement appropriate interventions and policies to alleviate them.

## Introduction

Cesarean section (C-section), also known as cesarean delivery, is performed through surgical incisions made in the abdomen and uterus to safely deliver a baby when vaginal delivery is considered risky for the mother or the baby [1]. When there are medical indications, a C-section can prevent maternal and perinatal morbidity and mortality [2]. In contrast, CS deliveries without a clinical need present risks to the mother and the neonate [3]. Although considered a life-saving technique, unlike any other surgical procedure, C-sections have a few short- and long-term negative health consequences for the mother and the baby [4]. For instance, abnormal placentation, stillbirth, ectopic pregnancy, and uterine rupture are a few risk factors contributing to maternal health. Babies, on the other hand, may experience adverse neonatal physiology through different physical, medical, hormonal, and bacterial exposures [4]. Moreover, the increased rate of CS also brings a financial burden to the family and the country’s health care system [5, 6]. Out-of-pocket expenses approximating USD 483 million dollars were paid out for medically unnecessary CS in 2018.

The acceptable rate of C-sections ranged from 10 to 15% according to a report by WHO [7] with a rate below 10% considered limited obstetric care and a rate above 15% indicating unnecessary use of the procedure [8]. However, the World has experienced an influx in C-section rates over the past few decades, and according to new research from World Health Organization (WHO), C-sections account for every 1 in 5 childbirths [9]. It is projected to increase in the coming decade and by 2030, approximately a third (29%) of all births are likely to be delivered by C-Sects. [9, 10]. Low- and middle-income (LMIC) countries are projected to see 33.5 million C-section deliveries by 2030 [10]. The rate of CS is expected to be risen to 63% for Eastern and 50% for Western Asian countries [10].

Approximately 42% of the CS deliveries worldwide are not medically indicated [11]. Contributing factors for unnecessary CS deliveries are the convenience of physicians, maternal preference, fear of pain, and childbirth over-medicalization [12]. Although a significant number of CS deliveries are considered unnecessary and avoidable [13], the rate of CS delivery in Bangladesh is one of the highest in the world (45% according to Bangladesh Demographic and Health survey (BDHS) 2022). Despite overwhelming increase, access to CS delivery is limited in rural setting due to high poverty rate, unavailability of equipped service facilities, and lack of health insurance coverage [11]. Therefore, progress towards achieving Sustainable Development Goals (SDGs) targets 3.1 (less than 70 maternal death per 100,000 live births), 3.2 (neonatal and under-five mortality as low as 12 and 25 per 1000 live births), and 3.7 (universal access to sexual and reproductive health coverage) by 2030 will be hindered if unnecessary deliveries are not restricted and universal access to necessary CS are not facilitated.

Available evidence manifested the presence of considerable inequalities in CS use both between and within countries [10]. Countries belongs to LMICs experiences double burden of CS – overuse and unmet need [10]. Inequalities in the use and access to the delivery care services are common and persistent among different socioeconomic sub-groups in LMICs [14]. Information on inequalities in CS use within a country across 72 LMICs are provided based on socio-economic status, such as, wealth quintiles and the place of residence [15]. CS use among the women who belongs to the richest wealth quintile are almost five times higher than that among the poorest wealth quintiles based on a study published in lancet series that studies 82 LMICs [16].

In Bangladesh, socioeconomic inequalities are well-documented across various indicators of maternal health care services [17–19]. CS use significantly varies based on age, education, wealth quintile, working status, and rural-urban residence [17, 20]. Approximately 7.5% of the women are deprived of availing CS deliveries for whom it was deemed necessary [13]. In addition, there are geographical diversities in Bangladesh where each areas manifest a unique characteristics and distinct forms of livelihood [21]. Annual average rate of increase in CS is experienced by South-western regions of Bangladesh compared to other geographical areas [20]. In an attempt to explore spatial distribution of CS deliveries, authors declared that Dhaka, Rajshahi, and Khulna divisions are hot spots due to high-level of CS deliveries, and Chattogram, Sylhet, Rangpur, and Mymenshingh divisions are cold spots due to low-level of CS deliveries [22]. Due to poor road condition and lack of transportations, remote areas lack accessibility to different health care services including obstetric care, especially during monsoon season [23]. Above all, delivery care service inequalities are projected to persist until 2030 according to a recent study [24].

There are no studies in Bangladesh that looked at both socio-economic and area-based disparities in CS deliveries using well-established rigorous approaches. Existing CS inequality studies in Bangladesh are either old [25, 26], not comprehensive [19], based on decomposition analysis [17, 27], or based on approaches not recommended by the WHO [17, 19, 25–27]. Use of recommended inequality analysis is necessary to overcome the limitation of previous studies in minimizing the gaps between sub-populations in terms of under or over utilization of CS deliveries and thus facilitating the SDGs targets. Therefore, this study aimed to investigate the inequalities and trend in CS use among Bangladeshi women using last two decades of demographic and health survey data from the year 2004 to 2017 using the World Health Organization (WHO) Health Equity Assessment Toolkit (HEAT) software. Findings from this study may facilitate the Government, stakeholders, and health care planners to design and implement intervention policies which will help in mitigating socioeconomic and geographical disparities in CS delivery care services in Bangladesh and other similar settings.

## Methods

### Study design and sampling

To measure the magnitude of inequality in the use of CS by women in Bangladesh in last two decades we used secondary data from BDHS from the year 2004 to 2017. The Demographic and Health Survey is conducted as a part of MEASURE program in the low- and middle-income countries and the data from the survey is stored in the Health Equity Assessment Toolkit (HEAT) software by WHO. In Bangladesh the demographic survey is conducted by National Institute of Population Research and Training (NIPORT) and the Ministry of Health and Family Welfare of Bangladesh partnered with the USAID which gives a nationally representative view. To conduct the DHS survey in Bangladesh a two-stage stratified cluster sampling technique is used. In the first stage enumeration area is selected from the whole country based on the last population census and is considered as the primary sampling unit for the survey. In the second stage, from the selected enumeration area households are selected for conducting the survey. The final report of the latest BDHS holds the details of the methodology for the sampling technique [28].

### Outcome variable

For this study, we used the CS use by women as delivery option in 3 years preceding the survey period [28] as the outcome variable. The response for the outcome variable was binary (yes or no). The women who delivered her last baby by CS was considered to have the response as yes. The response was coded as ‘1’ for ‘yes’ and ‘0’ for ‘no’.

### Equity dimensions

Four inequality indicators namely wealth quintile, education, place of residence and sub-national region were used to measure the CS use by women in Bangladesh. Wealth quintile was categorized in 5 categories as poorest, poorer, middle, richer, and richest as a composite variable deriving from 3 different variables according to the principal component analysis (PCA) technique [29]. Highest level of educational attainment was measured as education sub dividing as no schooling, primary, and secondary / higher [30]. Urban and rural residence was captured as the place of residence whilst the administrative divisions of Bangladesh was considered as the sub-national region.

### Statistical analysis

The latest version of the HEAT software by WHO was used to measure the inequality in the CS use over the last two decades among the women in Bangladesh [31]. The prevalence of CS by demographic variables over the years, along with their 95% confidence intervals, was computed. Estimated Annual Percentage Change (EAPC) was also reported to observe average annual percentage change in prevalence over a fixed time interval. EAPC was measured using a linear regression-based method proposed by Hankey [32].

To measure the magnitude of the inequality, we used four measures named Difference (D), Population Attributable Fraction (PAF), Population Attributable Risk (PAR), and Ratio (R). Of these four measures, D and R are the simple unweighted measures, and PAF, PAR are the complex weighted measures. Simultaneously, D and PAF are the absolute measures, and R and PAR are the relative measures. Out of all the absolute and relative summary measures available in the software, only these four measures (D, PAF, PAR, and R) were used to estimate the inequalities, since these are applicable for both order and non-ordered variables [33]. The choice of the summary measures of both absolute and relative was considered according to the recommendation of WHO [34], which dictates the importance of both absolute and relative measures to generate a finding that is policy driven [31]. Unlike the simple measures, the complex measures are weighted measures that take into account the situations in each population subgroup, and may also take the population share of each subgroup into consideration [33]. The elaborate technique used to generate the summary measures are extensively described by WHO elsewhere [34, 35].

In order to calculate the inequalities of the ordered variables like wealth quintile and educational level, D is calculated as the difference between the highest and the lowest category. For example, D for the wealth quintile is calculated by subtracting the prevalence of poorest group from the richest group. Again, R is calculated as the ratio between the highest and lowest group. For instance, R for the educational level is the division of the prevalence of the secondary or higher educational group by the no schooling group. Whereas in the case of the non-ordered variable like place of residence and sub-national region, the group with the highest prevalence is considered as the reference group, and D is measured by subtracting the group with the lowest prevalence from the group with the highest prevalence. Likewise, R is calculated by dividing the group with highest prevalence by the group with the lowest prevalence [36].

The calculation of complex measures like PAF and PAR are bit different from the simple measures. For the calculation of the PAF and PAR population mean is required. For our calculation, we considered the national average ($\mu )$ as the population average. The PAR is calculated as the difference between the most advantageous subgroup (group with the highest prevalence) and the national average $\mu$. PAR = Y_ref_ – $\mu$; since our calculation did not have a defined reference category the most advantageous group was considered as the reference category. From the PAR, PAF is calculated by dividing the PAR by the national average and multiplying the result by 100. PAF = [(PAR / $\mu$) × 100] [30, 37]. The details of the calculation are described in the technical notes by WHO [38]. For all four measures of inequality, both simple and complex, the higher values of the measure indicate higher inequality. The values in the positive direction indicate the inequality favors the advantageous group, whilst the values in negative direction indicate the inequality favoring the disadvantageous group [33]. To measure the significance of the inequality measures, we calculated the 95% confidence interval along with point estimates for each of the four measures. The persist inequalities for D, PAF, and PAR were found significant if the confidence interval does not contain 0, while for R, the absence of 1 in the confidence interval was considered as significant inequality.

## Results

### Prevalence of C-section across equity dimensions

**Fig. 1:**
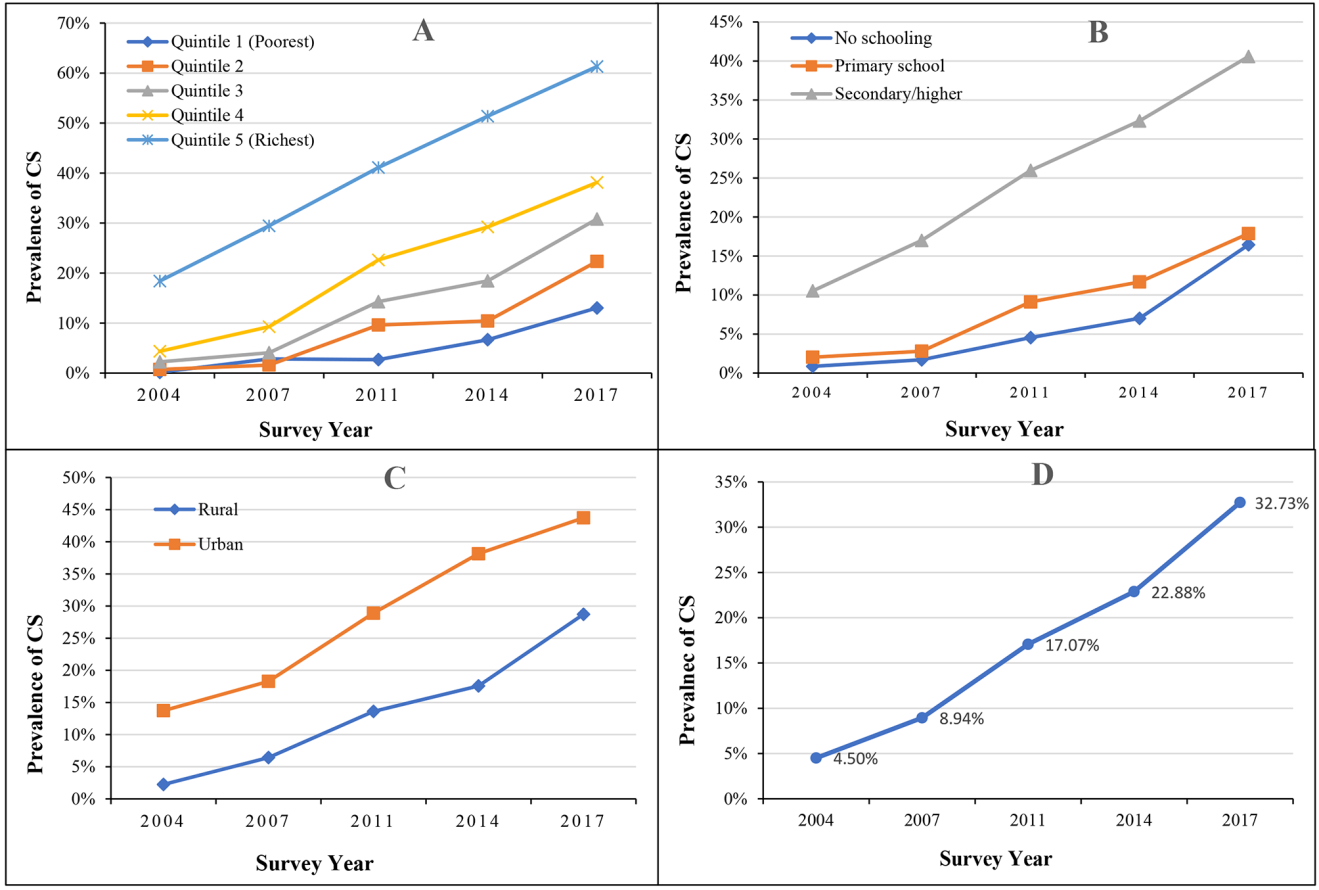
Trend and prevalence of CS in Bangladesh from 2004 to 2017. (**A**) denotes the CS in Bangladesh based on household wealth quintile from 2004 to 2017. (**B**) shows the CS in Bangladesh based on education level of women from 2004 to 2017. (**C**) shows the CS in Bangladesh based on place of residence from 2004 to 2017. (**D**) demonstrates the trend in the prevalence of CS in Bangladesh from 2004 to 2017

Overall, an increasing trend in the prevalence of C-section was found from 2004 to 2017, with 4.50% in 2004, 17.07% in 2011, and 32.73% in 2017 (Fig. 1 (D)). A rising trend in using C-section was observed among all the sub-groups of equity dimensions. For instance, women from poorest and richest wealth quintile showed a prevalence of 0.15% and 18.40% in 2004 which increased to 13.03% and 61.30%, respectively, in 2017 (Fig. 1 (A)).Regarding the educational qualification of women, those having no schooling/no formal education had a lower prevalence of CS in all survey rounds (from 0.86% in 2004 to 16.44% in 2017), while those who had secondary or higher education showed a higher prevalence of CS with 10.53% in 2004 to 40.58% in 2017 (Fig. 1 (B)). A rural-urban gap was observed in the prevalence of C-section, with higher prevalence in urban areas in all the survey points (i.e., 28.72% in rural areas and 43.72% in urban areas in 2017) (Fig. 1 (C)). Based on the administrative divisions of Bangladesh, geographical differences in the prevalence of C-section were also found, with Khulna having the highest prevalence followed by Dhaka division in all survey points except in 2004. For example, in 2017, Khulna showed a prevalence of 42.71% followed by Dhaka (42.69%), while the lowest prevalence was found in Sylhet (22.63%) division (Table 1).

**Table 1:** Trends in the prevalence of cesarean delivery, disintegrated across four inequality dimensions, from years 2004 to 2017

Inequality Dimension	2004 (4.50%)		2007 (8.94%)		2011 (17.07%)		2014 (22.88%)		2017 (32.73%)		EAPC (95% CI)
	n	Estimate (95% CI)	n	Estimate (95% CI)	n	Estimate (95% CI)	n	Estimate (95% CI)	n	Estimate (95% CI)	
**Economic Status**											
Quintile 1 (poorest)	1029	0.15 (0.02–1.05)	758	2.82 (1.59–4.97)	1135	2.69 (1.84–3.91)	1084	6.67 (4.41–9.98)	1108	13.03 (10.62–15.89)	34.13 (14.20-57.54)
Quintile 2	831	0.72 (0.31–1.64)	779	1.60 (0.82–3.11)	1003	9.61 (7.27–12.59)	932	10.42 (8.16–13.22)	1106	22.33 (19.25–25.73)	30.69 (21.74–40.30)
Quintile 3	848	2.24 (1.15–4.32)	699	4.06 (2.57–6.35)	974	14.28 (11.85–17.10)	942	18.44 (14.93–22.54)	1020	30.81 (27.31–34.55)	22.93 (17.95–28.12)
Quintile 4	717	4.35 (2.97–6.33)	694	9.27 (6.78–12.57)	963	22.62 (19.53–26.05)	995	29.22 (25.34–33.42)	1071	38.12 (34.62–41.74)	18.25 (13.20-23.52)
Quintile 5 (richest)	695	18.40 (15.54–21.66)	659	29.45 (25.64–33.57)	881	41.11 (37.21–45.13)	950	51.37 (47.31–55.41)	1034	61.30 (57.68–64.81)	9.41 (7.36–11.50)
**Level of Education**											
No schooling	1463	0.86 (0.52–1.41)	856	1.70 (1.01–2.85)	892	4.54 (3.19–6.44)	704	7.02 (4.90–9.95)	351	16.44 (12.20-21.78)	24.85 (22.53–27.21)
Primary School	1256	2.03 (1.19–3.44)	1103	2.80 (1.89–4.13)	1485	9.12 (7.22–11.45)	1380	11.68 (9.51–14.28)	1471	17.88 (15.47–20.58)	19.35 (14.82–24.06)
Secondary / Higher	1400	10.53 (9.07–12.19)	1619	17.00 (14.84–19.41)	2579	25.98 (23.87–28.20)	2820	32.32 (29.81–34.94)	3515	40.58 (38.35–42.84)	10.68 (8.66–12.75)
**Place of Residence**											
Rural	3307	2.24 (1.72–2.92)	2828	6.42 (5.28–7.80)	3835	13.61 (12.00-15.41)	3637	17.57 (15.47–19.88)	3911	28.72 (26.43–31.13)	20.39 (14.68–26.38)
Urban	813	13.72 (11.18–16.73)	761	18.28 (15.20-21.81)	1121	28.89 (25.60-32.42)	1267	38.14 (34.03–42.42)	1427	43.72 (40.63–46.87)	9.75 (8.27–11.25)
**Sub-National Regions**											
Barishal	237	3.14 (1.90–5.15)	219	4.13 (2.21–7.59)	273	13.16 (9.45–18.03)	279	17.72 (12.54–24.44)	303	24.55 (20.03–29.71)	18.60 (13.95–23.44)
Chattogram	920	2.78 (1.84–4.18)	798	7.58 (5.65–10.09)	1176	13.95 (11.33–17.06)	1074	18.33 (14.79–22.51)	1141	26.05 (21.91–30.66)	17.67 (12.16–23.45)
Dhaka	1250	7.27 (5.66–9.30)	1132	11.95 (9.40-15.07)	1510	20.25 (16.87–24.10)	1740	29.14 (24.48–34.29)	1359	42.69 (38.00-47.51)	14.38 (13.40-15.37)
Khulna	450	5.30 (3.69–7.55)	328	12.23 (9.05–16.33)	463	26.25 (21.72–31.35)	387	33.00 (28.40-37.94)	481	42.71 (37.46–48.13)	17.06 (11.82–22.55)
Mymenshingh	-	-	-	-	-	-	-	-	451	26.08 (21.93–30.70)	
Rajshahi	896	2.96 (2.05–4.27)	792	7.40 (5.35–10.15)	646	17.64 (13.77–22.33)	488	22.26 (18.15-27.00)	622	35.61 (30.31–41.29)	20.31 (15.17–25.69)
Rangpur	-	-	-	-	513	11.62 (8.86–15.09)	461	17.50 (13.62–22.20)	555	27.85 (22.62–33.76)	15.68 (14.49–16.89)
Sylhet	366	3.07 (2.03–4.62)	319	5.38 (3.83–7.52)	375	12.00 (9.12–15.63)	474	10.87 (8.05–14.52)	425	22.63 (18.02–28.02)	15.37 (10.60-20.33)

CI: Confidence Interval, EAPC: Estimated Annual Percentage Changes

Mymensingh division was separated from Dhaka division in 2015, and Rangpur division was separated from Rajshahi division in 2010. Hence, the estimates for BDHS 2004 to 2014 data of Mymensingh, and BDHS 2004 to 2007 data of Rangpur division are not shown in the table

We have also incorporated the Expected Annual Percentage Change (EAPC) values in the final column of Table 1. It was observed that regardless of economic status the CS prevalence increased over the years, however the average annual percentage change was gradually higher from the richest (34.13%/year) to the poorest (9.41%/year). EAPC values were comparatively lower for the women with higher educational status (24.85%/year for no schooling to 10.68%/year for secondary and higher). The average annual increase in prevalence in CS from 2004 to 2017 were approximately half for the Urban residents (9.75%) as compared to the Rural residents (20.39%). Approximately a similar average annual increase in CS was observed across administrative divisions (Table 1).

### Disparities in C-section over time

Socio-economic inequalities based on economic status and educational qualification of women as well as geographical inequalities based on place of residence and administrative division were identified. Based on both absolute (D and PAF) and relative (R and PAR) measures, wealth-related disparities were obtained favoring the richest sub-group. For instance, the R value of 4.70 (95% CI: 3.82–5.80) in 2017 indicates a significant disparity with the higher use among richest group compared to the poorest group. This wealth-driven disparity was found in all survey points, though it was reduced from 2004 to 2017. Disparity based on the women educational level was also observed in all survey waves with higher concentration among those having higher education. For example, the PAF measure of 23.97 (95% CI: 12.26–35.68) in 2017 demonstrates a significant inequality with higher prevalence among those having secondary / higher education (Table 2).

**Table 2:** Inequality indices estimates of prevalence of cesarean delivery in Bangladesh, years 2004–2017

Inequality Dimension	2004		2007		2011		2014		2017	
	Estimate	95% CI	Estimate	95% CI	Estimate	95% CI	Estimate	95% CI	Estimate	95% CI
**Economic status**										
D	18.26	15.21–21.31	26.63	22.37–30.89	38.43	34.34–42.51	44.70	39.81–49.58	48.27	43.85–52.70
PAF	308.62	300.99-316.25	229.57	216.50-242.64	140.87	135.37-146.38	124.48	118.19-130.77	87.28	81.52–93.05
PAR	13.90	13.56–14.24	20.51	19.35–21.68	24.04	23.11–24.98	28.49	27.05–29.92	28.57	26.69–30.46
R	125.76	17.54-901.52	10.43	5.82–18.70	15.30	10.38–22.55	7.70	5.08–11.67	4.70	3.82–5.80
**Level of Education**										
D	9.67	8.06–11.27	15.31	12.88–17.74	21.43	18.75–24.11	25.31	21.73–28.88	24.14	18.88–29.40
PAF	133.78	123.16-144.39	89.73	79.92–99.54	52.19	44.29–60.09	41.25	33.12–49.37	23.97	12.26–35.68
PAR	6.03	5.55–6.50	8.04	7.16–8.92	8.91	7.56–10.26	9.44	7.58–11.30	7.85	4.01–11.68
R	12.23	7.31–20.46	10.03	5.88–17.11	5.72	3.99–8.20	4.61	3.20–6.62	2.47	1.84–3.31
**Place of Residence**										
D	11.48	8.67–14.30	11.86	8.35–15.37	15.28	11.47–19.09	20.57	15.84–25.31	15.00	11.10–18.90
PAF	204.69	196.03-213.34	104.54	97.99-111.09	69.28	65.47–73.09	66.68	63.28–70.07	33.58	31.12–36.03
PAR	9.22	8.83–9.61	9.34	8.76–9.93	11.82	11.17–12.47	15.26	14.48–16.03	10.99	10.19–11.79
R	6.13	4.40–8.54	2.85	2.18–3.71	2.12	1.79–2.52	2.17	1.84–2.56	1.52	1.37–1.70
**Sub-National Region**										
D	4.49	2.37–6.62	8.11	3.71–12.50	14.63	8.91–20.35	22.13	16.38–27.88	20.08	12.77–27.39
PAF	61.43	39.11–83.75	36.89	5.90-67.89	53.79	38.02–69.56	44.21	32.16–56.26	30.48	18.66–42.30
PAR	2.77	1.76–3.77	3.30	0.53–6.07	9.18	6.49–11.87	10.12	7.36–12.87	9.98	6.11–13.85
R	2.62	1.62–4.22	2.96	1.50–5.86	2.26	1.64–3.12	3.04	2.19–4.21	1.89	1.46–2.43

CI: Confidence Interval, D: Difference, PAR: Population Attributable risk, PAF: Population Attributable Fraction, R: Ratio

Significant rural-urban disparities were also obtained over time using both simple (D, R) and complex (PAF, PAR) inequality measures with higher concentration in urban areas. With the time, the rural-urban disparity was decreased. For instance, the PAF measure of 204.69 (95% CI: 196.03–213.34) in 2004 was reduced to 33.58 (95% CI: 31.12–36.03) in 2017. When looking at the sub-national regions (administrative divisions of Bangladesh), a higher concentration of C-section in Khulna and Dhaka divisions was observed and significant regional disparities was obtained disfavoring the Sylhet division. This inequality was reflected by the PAF of 30.48 (95% CI: 18.66–42.30) as absolute measure and PAR of 9.98 (95% CI: 6.11–13.85) as relative measure in 2017 (Table 2).

## Discussion

In our study, the prevalence of cesarean section has been found with an increasing trend from 4.50% in 2004 to 32.73% in 2017. There has been a 751% rise in the use of CS in Bangladesh over the last two decades [13]. Similar increasing trend was found previously in a study conducted in Bangladesh [13]. This upward rising in the prevalence of CS in Bangladesh could be due to several reasons. Firstly, the growing number of private health sectors in all over Bangladesh [39] and their propensity to use CS with higher tendency of providing incentive to the physicians to motivate them for advising CS [40, 41]. Besides many physicians find it to be time effective than the normal delivery [20, 41]. Secondly, fear of pain in vaginal delivery [42, 43] and false sense of better quality care by CS [44]. could be another reason behind increasing prevalence of CS in Bangladesh over time. Thirdly, insufficiency of the support and convenience from both side (provider and receiver) in terms of vaginal delivery could also contribute to the increasing prevalence of CS [40]. Lastly, lifestyle change in women due to rapid urbanization leading to obesity has made them more vulnerable to complication during pregnancy and delivery [45] and to avoid them CS prevalence has substantially increased in Bangladesh over time.

Over the years in the last two-decade people in the poorest quintile has been found consistently to have the lowest prevalence of CS use compared to the people in richest quintile. Despite the increase in the prevalence of CS in all wealth quintile the rise in the prevalence in the poorest quintile was found significantly small than the high rise of CS among the richest quintile people. This result corroborates with the finding of the studies conducted previously in Bangladesh [17, 20, 46], India [12, 47, 48], Nigeria [49], Ghana [35], and Burundi [50]. The possible reasons behind this finding could the women in higher wealth quintile prefer to have the delivery by CS as a safe option being able to bear the expenses [51]. Also rich women are more likely to use the private facility which increases the possibility of having the baby by CS [51]. Again women from higher wealth quintile usually more acquainted with comfort and facility and less likely to embrace the pain from vaginal delivery [52]. Another reason behind this finding could be that women in higher health quintile are more autonomous in taking their health care decision making [53].

Women with secondary or higher educational attainment was found to be using CS higher than the women with no education. The increasing trend of CS use among women with the increase in education has been found consistent over the years. This finding coincides with the result conducted previously in Bangladesh [17, 20], India [48], Pakistan [54], China [55], Nepal [56], Nigeria [49], and Ghana [35]. The possible explanation behind this finding could be educated women are more like to belong to the higher economic status which contribute to the selection of private facility for delivery leading to increased number of CS [48]. Again educated women tends to take their own healthcare decision resulting in choosing CS as perceived safe and painless option for delivery [53]. Higher educated women in Bangladesh has higher rate of obesity and delayed pregnancy which facilitates pregnancy complications necessitating CS as delivery option [45]. Women with higher education are also more concerned about their vaginal appearance and to preserve the aesthetic usually prefer CS over the vaginal delivery [57, 58].

Our study found that women living in the urban areas are more likely to use CS as delivery option compared to the rural women in all the survey years. Although the prevalence of cesarean section delivery increased in both urban and rural settings the rate of increase in much higher in the urban areas than the rural areas. Studies conducted in Bangladesh [17, 20], India [47, 48], Nepal [56], Nigeria [49], and Burundi [50] supports this finding. The feasible explanation behind this finding could be the higher availability [52] and better accessibility [59] of the heath facility in the urban area than the rural area. Besides the women living in the urban area are more likely to be educated and belonging to wealthier families, both of which attribute in the preference of CS over normal delivery [40, 60]. Women of urban areas have also been found to have sedentary lifestyle and in higher risk of obesity and its related complication [49]. This could also act as another contributing factor in increased prevalence of CS among the urban women.

Difference in the prevalence of CS has been observed among the sub national region in all survey years over the last two decades. Although subsequent increase in the prevalence of CS use was found in all the administrative division of Bangladesh over time, Dhaka and Khulna was found to have the highest prevalence of CS use among all the sub national regions and Sylhet and Barisal was found to have the lowest prevalence. Similar results were also found in previous studies in Bangladesh [20, 22]. One of the potential reason behind this finding could be the difference in the knowledge of the risk about unnecessary CS and advantages of vaginal delivery among different regions in Bangladesh [61]. The availability of the health facility in different region may differ along with the cultural belief could also play an important role in the use of CS among different regions. Again the literacy rate in Dhaka and Khulna was found much higher than the Sylhet and Barisal [62] which could be another reasonable cause of higher prevalence of CS in Dhaka and Khulna compared to other sub national regions.

### Strengths and limitations

The use of nationally representative data over the last two decades has made the result of this study generalizable to all the women in Bangladesh and also makes it easy to understand the pattern of change in the prevalence of CS use among women. In this study, we used both absolute and relative measure to assess the magnitude of inequality of CS use among the women which simultaneously satisfy the WHO criteria for inequality measurement and also gives a multi-dimensional view of the situation. We also considered wealth quintile and education as the socioeconomic dimensions and place of residence and sub national region as the geographic dimensions providing a wider view in the inequality of CS use among the women of Bangladesh. Lastly the use of WHO’s HEAT software makes our result more accurate, reliable, and appropriate. This study also poses with some limitations. Since we used secondary cross-sectional data to measure the inequalities, we could not identify the causes of inequality. The surveys are also prone to recall and reporting bias that could not be overlooked. Due to the statistical analysis technique and unavailability of the variables in the data sets, we could not consider other important dimensions like social and cultural dimensions in the study. Also, the built-in version of the HEAT software does not include many socioeconomic variables like GDP, family income, occupation which limits the comprehensiveness of the inequality measures based on the socioeconomic dimension.

### Public health and policy implications

This study might have some public health and policy implications where policies should be designed to reduce the inequalities in CS in Bangladesh. Comprehensive health education and awareness campaigns regarding the consequences of CS are required, according to the observed socioeconomic inequalities in CS in Bangladesh. The goal of these initiatives should be raising awareness among women from urban areas with higher socioeconomic status about the complications and consequences of CS for both mothers and child. Thus, policies should focus on the empowered women with higher socioeconomic status to decline the prevalence of CS in this group and the reduce the disparities. Examples of this include enacting social protection programs for underprivileged women and advocating for equal access to work and education. Policies have to focus on expanding awareness programs to avoid unnecessary CS, especially women from urban areas and Khulna division.

## Conclusion

The study revealed that there remain significant inequalities in both socioeconomic and geographic dimensions in the use of CS among the women in Bangladesh. Women belonging to the rich quintile, attaining higher education, living in the urban area were found advantageous in all the survey years in the last two decades. Besides the Khulna was found to have the highest and Sylhet to have the lowest prevalence in CS use over the years. Further longitudinal studies are warranted to find out the cause of inequality in the use of CS. Policymakers should pay special attention to the disadvantageous group in ensuring their use of CS when necessary.

## Data Availability

The study used data from the Bangladesh Demographic and Health Survey 2004, 2007, 2011, 2014 and 2017-18. The data sets are available at: https://dhsprogram.com/data/available-datasets.cfm.

## Abbreviations

CS: Cesarean Section
WHO: World Health Organization
HEAT: Health Equity Assessment Toolkit
BDHS: Bangladesh Demographic and Health Survey
SDG: Sustainable Development Goals
LMIC: Low-and-middle income country
CI: Confidence Interval
PAF: Population Attributable Fraction
PAR: Population Attributable Risk
PCA: Principal Component Analysis
